# Postoperative Ileocolic Intussusception in a Neonate with Anorectal Malformation

**DOI:** 10.21699/ajcr.v7i5.473

**Published:** 2016-11-01

**Authors:** Piyush Kumar, Sudhir Singh, J D Rawat, Sarita Singh

**Affiliations:** 1 Department of Pediatric Surgery, King George’s Medical University, Lucknow, India; 2 Department of Anesthesia, King George’s Medical University, Lucknow, India

**Dear Sir,**

Neonatal intussusception is relatively rare entity with an incidence of 0.3% of all intussusception.[1] Rarer still are postoperative intussusceptions in the same age group.[2-4] We report a case of postoperative intussusception in a neonate with anorectal malformation.

A 7-days-old male neonate with anorectal malformation (ARM) presented with abdominal distension and bleeding per rectum. He had an operation for ARM (Anoplasty) a day after birth and the patient had history of passing stool normally for two days. It was followed by blood mixed stool and abdominal distension. On examination, the patient had features of mild dehydration and sepsis. The abdomen was distended and a mass was palpable on left side of abdomen. Perineal examination showed attempt of a primary anoplasty performed at other hospital for absent anal opening to decompress the bowel. Haematological and biochemical investigations including coagulation profile were in the normal limits. X-ray abdomen in AP erect view revealed multiple air fluid levels. Patient was resuscitated with intravenous (IV) fluids. Antibiotics and analgesics were started. In view of abdominal distension, palpable abdominal mass, and history of bloody stool, we kept the possibility of intussusception in mind. Exploratory laparotomy was performed after about four hours of admission. Intraoperatively, ileocolic intussusception was found, which was advancing up to the sigmoid colon (Fig.1). It was not possible to manually reduce the intussusception owing to ischemic intussusceptum, thus resection of bowel with ileostomy and colonic mucous fistula (Transverse colon) were made. On opening the resected bowel, no pathological lead point was found. Patient was discharged in good general condition on fifth post-operative day of procedure. Sitz bath and care of perineal wound was advised. Patient was in regular follow up or two months with satisfactory condition. However, he did not turn up in third month. On telephonic inquiry, it was informed that the patient developed ileostomy diarrhoea and succumbed to dehydration.

**Figure:**
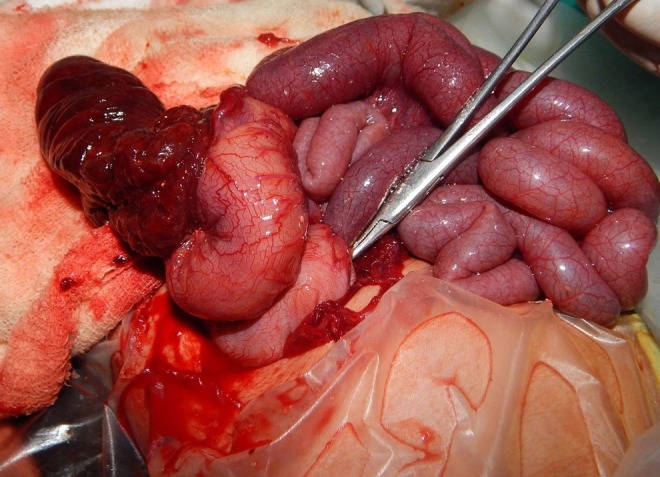
Figure 1: Ileocolic intussusception. The colon has ruptured while attempts of reduction showing devitalized intussusceptum.

Neonatal post-operative intussusception is very rare entity and only few cases are reported in literature. It has been reported after operations for duodenal stenosis, malrotation of gut, cutis laxa and hiatal hernia, ARM, eventration of diaphragm, patent vitellointestinal duct etc.[2-4] In our case it developed after anoplasty for ARM. Diagnosis of the post- operative intussusception at any age is very difficult in general and very challenging in the neonatal period. A delay in diagnosis and management in neonates may lead to more serious complications such as septicemia compared to older children. In our case, we had suspicion of postoperative intussusception and early intervention led to reasonable early outcome. Unfortunately, the child died due to complications of high stoma output.

## Footnotes

**Source of Support:** Nil

**Conflict of Interest:** None declared

